# Common Microbial Genital Infections and Their Impact on the Innate Immune Response to HPV in Cervical Cells

**DOI:** 10.3390/pathogens11111361

**Published:** 2022-11-16

**Authors:** Matteo Fracella, Giuseppe Oliveto, Leonardo Sorrentino, Piergiorgio Roberto, Lilia Cinti, Agnese Viscido, Federica Maria Di Lella, Federica Giuffrè, Massimo Gentile, Valeria Pietropaolo, Carla Prezioso, Ettore Palma, Nadia Recine, Innocenza Palaia, Carolina Scagnolari, Guido Antonelli, Alessandra Pierangeli

**Affiliations:** 1 Laboratory of Microbiology and Virology, Department of Molecular Medicine, Sapienza University of Rome, 00185 Rome, Italy; 2 Microbiology and Virology Unit, Sapienza University Hospital Policlinico Umberto I, 00161 Rome, Italy; 3 Department of Public Health and Infectious Diseases, Sapienza University of Rome, 00185 Rome, Italy; 4 Department of Maternal Infantile and Urological Sciences, Sapienza University of Rome, 00185 Rome, Italy

**Keywords:** human papillomavirus, genital infections, vaginosis, sexually transmitted infections, type III interferon, TLR9, CCL20

## Abstract

The persistence of high-risk (HR) human papillomavirus (HPV) genotypes is a prerequisite of cervical cancer. It is not clear whether and how bacterial vaginosis (BV) and sexually transmitted infections (STIs) cause higher rates of persistent HPV infection. This study aimed to characterize mucosal innate immunity to HPV, comparing different conditions. Specifically, expression levels of genes coding for Toll-like receptors (TLR)7 and 9, several type III Interferon-related genes (IFNL1, 2, 3, their specific receptor subunit IFNLR1, and the IFN-stimulated gene ISG15). Chemokines CCL5 and CCL20 were measured in cervical cells positive, or not, for HPV, BV, and STIs. HPV DNA was detected in 51/120 (42.5%) enrolled women, two/third were HR-HPV genotypes. More than 50% of samples were BV- and/or STI-positive. HPV-positive women had BV, but not other STIs, more frequently than the HPV-negative. TLR9 and IFNL1 mRNAs were expressed in the LR, but much less in the HR HPV infection. Enhanced levels of TLR9, TLR7, IFNL2, and IFNLR1 were observed in HPV-positive women with BV and STI. TLR9-increased expression was associated with HPV persistence in previous studies; hence, bacterial coinfections may enhance this risk. Prospective measurements of type III IFNs and IFNLR1 are warranted to evaluate whether this response may act as a double-edged sword in infected epithelia.

## 1. Introduction

Human papillomavirus (HPV) is a small, non-enveloped, and double-stranded DNA virus responsible for the most common sexually transmitted infection [1]. Mucosal low-risk (LR) HPV genotypes cause benign lesions while high-risk (HR) genotypes are associated with cervical cancer [2]. HPV genotypes have been recently classified by the IARC [3] into three groups according to their oncogenic risk. Most HPV infections are cleared in a period between 6 and 18 months by the host immune system, but a small fraction of HR-HPV can establish persistent infections that are associated with cancer risk [4]. The factors that lead to HPV persistence are not yet well understood.

In the mucosal response to pathogens, Toll-like receptors (TLR) play a central role in leading to the production of interferons (IFNs) and inflammatory cytokines, an unbalanced or prolonged TLR activation may act against the local commensal microflora and/or cause inflammatory tissue damages [5,6]. Particularly important in female genital mucosa is the expression of TLR9 which recognizes microbial unmethylated double-stranded DNA CpG motifs [5,6]. TLR7 mainly recognizes RNA but its activation has been related to cervical HPV clearance [7].

Type I IFNs are essential for host defense against viruses and bacteria through the induction of antimicrobial effector molecules that are encoded by IFN-stimulated genes. HR-HPVs downregulate the expression and signaling of type I IFNs by different molecular mechanisms [4]; nonetheless, type I IFNs are activated in the first phases of HPV natural infections [7,8]. Type III IFNs, also known as IFN lambda (IFNL) 1–4, are primarily produced and active in epithelial cells, due to the restricted expression of their receptor composed by the subunits IFNLR1 and IL10R2 [9]. Little is known about the role of the IFNLs during HPV infections, in our previous study, we demonstrated a lower IFNL1 expression in cervical cells from HR-HPV-positive with respect to LR-HPV-positive women [10].

In response to danger signals, keratinocytes readily upregulate the production and secretion of proinflammatory chemokines, thus activating a cell-mediated response [11]. In particular, the C-C motif ligand (CCL) 20 is necessary for the recruitment of Langerhans cells, which coordinate the first defense responses to infection in epithelial tissues [12]. However, HPV oncoproteins can interfere with the expression of CCL20 and CCL5 to establish prolonged infections [13,14]. No previous studies measured these important cytokines in the first phases of HPV infections in cervical cells.

Besides HPV, several microorganisms can infect the cervicovaginal tract causing a wide range of clinical conditions from completely asymptomatic to severe [15]. While sexually transmitted infections (STIs) caused by *Neisseria gonorrhoeae*, *Chlamydia trachomatis*, and *Trichomonas vaginalis* are relatively well characterized, the pathogenic role of other microbes, such as *Mycoplasma genitalium*, *Candida* species (spp.), and *Ureaplasma* spp.—which often occur in multiple infections and with no or aspecific symptoms— is not well defined [15] (ref). STIs are increasingly common [16] but underdiagnosed in the clinical context [15]. Recently developed multiplex-PCR assays may contribute to better assessing the epidemiology of specific STIs and their clinical relevance in relation to women’s age and other genital conditions [17,18]. Bacterial vaginosis (BV) represents an important determinant influencing STIs’ prevalence and persistence. BV is a clinical condition caused by alterations in the vaginal microbiota (dysbiosis) that, from a predominance of the protective *Lactobacillus* spp. [19], shifts to a higher abundance of anaerobic bacteria, mainly *Gardnerella*, *Prevotella,* and *Atopobium* spp. but also *Bacteroides fragilis*, *Mobiluncus* spp., *Megasphaera* Type 1, and others [20,21]. Although BV can be unapparent, it is often characterized by symptoms (e.g., vaginal discharge, “fish” odor). BV increases the risk of contracting STIs, such as *N. gonorrhoeae*, *C. trachomatis*, *T. vaginalis*, and those caused by viruses such as herpes simplex virus-2 (HSV-2), HIV, and HPV [22,23,24,25,26,27], likely due to a deregulated cervicovaginal innate immune response [28,29].

Although STIs and BV were temporally associated with persistent HPV infection [24,26,30,31], the influence of microbial species on the antiviral immune response has not been fully defined. In particular, it is not clear which target genes of the antiviral and inflammatory response, and to which extent, are influenced by the concomitant presence of BV and other STIs. To clarify these issues, we sought to analyze the innate immune response in genital mucosa by comparing selected women samples on the base of different infectious conditions. Specifically, we measured transcript levels of TLRs 7 and 9, several type III IFN-related genes (IFN lambda 1, 2, and 3, IFNLR1, and the IFN-stimulated gene ISG15), and the chemokines CCL5 and CCL20 in cervical cells in response to HPV, in the presence or lack thereof of BV and other STIs. The TLR9 and IFNLR1 increased expression observed in HPV and bacterial coinfections could enhance the risk of HPV persistence and oncogenic progression.

## 2. Materials and Methods

### 2.1. Study Group and Sample Collection

Between January 2018 to December 2019, women attending the cervicovaginal pathology unit of “Sapienza” university hospital were enrolled. To minimize confounding factors related to the resident microbial composition of the cervicovaginal mucosa, strict inclusion criteria were adopted: Italian nationality and ancestry, between 18 and 48 years of age (y), sexually active, not in pregnancy, with an intact uterus, not in menopause, in the pre-ovulatory phase, negative to HIV and HSV-2, no previous treatment for HPV-related lesions, no use of vaginal medication in previous 3 days, and no use of probiotics in previous 6 months. Patients’ cervical cells were collected with a cytobrush from both ectocervix and endocervix and added to 5 mL preservCyt solution for subsequent analyses: bacterial vaginosis (BV) diagnosis; HPV detection and genotyping; BV-associated bacteria and other STI pathogens molecular detection and gene expression quantification. Deidentified data were collected after informed consent was signed, and the study was approved by the Policlinico Umberto I Hospital Review Board and Ethics Committee (Rif. 4990, Prot. V3.0/2018).

### 2.2. HPV DNA Detection and Genotyping

Cervical cells were centrifuged at low speed, cells pellet was used for DNA extraction using a QIAamp Blood & Tissue Kit (Qiagen, Hilden, Germany). Purified DNA was subjected to a polymerase chain reaction (PCR) targeting human leucocyte antigen (HLA) in order to assess the efficacy of nucleic acid extraction. A 450 bp fragment from the L1 HPV region was amplified by the widely used primers MY09 and MY11, and the genotype was obtained by amplicon sequencing [15]. Samples with mixed sequencing chromatograms were excluded from further analysis. HPV genotypes were classified as HR (also including the probable HR 53 and 66) or LR according to Munoz et al. [2]. Alternatively, HPV-positive samples were stratified according to the more recent IARC classification [3] in three groups: carcinogenic to humans, group 1 (HPV 16, 18, 31, 33, 35, 39, 45, 51, 52, 56, 58, and 59), possible and probable carcinogenic, group 2A and 2B (HPV 26, 30, 34, 53, 66, 67, 68, 69, 70, 73, 82, 85, and 97); not classifiable as carcinogenic, group 3 (HPV 6, 11, 28, 32, 40, 42, 43, 44, 54, 55, 57, 61, 62, 71, 72, 74, 81, 83, 84, 86, 87, and 89).

### 2.3. BV- and STI-Detection

A diagnosis of bacterial vaginosis (BV) was given by the clinicians according to a microscopical examination of Gram-stained bacteria and determination of the Nugent score [32], a score of 7 to 10 was considered consistent with BV. Multiplex Real-time (RT) PCR assays (Allplex™ STI/BV Panel Assays, Seegene, Seoul, Republic of Korea) were performed, according to the manufacturer’s instruction, to identify the following STI pathogens: *Chlamydia trachomatis* (CT), *Neisseria gonorrhoeae* (NG), *Mycoplasma genitalium* (MG), *M. hominis* (MH), *Ureaplasma urealyticum* (UU), *U. parvum* (UP), *Thrichomonas vaginalis* (TV) (Panel 1); *Candida albicans*, *C. glabrata*, *C. tropicalis*, *C. parapsilosis*, *C. krusei*, *C. lusitaniae*, and *C. dubliniensis* (Panel 3); and the BV-associated bacteria: *Lactobacillus* spp. (Lacto), *Gardnerella vaginalis* (GV), *Atopobium vaginae* (AV), Bacterial vaginosis–associated bacteria 2 (BVAB2), *Bacteroides fragilis* (BF), *Mobiluncus* spp. (Mob), and *Megasphaera* Type 1 (Mega1) (Panel 4). On the basis of BV-associated bacteria detection, BV was diagnosed according to the following criteria: prevalence of *Lactobacillus* spp. with undetectable BV-associated bacteria was considered BV negative; *Lactobacillus* spp. levels still predominant in the presence of BV-associated bacteria were considered intermediate BV; the presence of high levels of BV-associated bacteria was considered BV positive.

### 2.4. Gene Expression Quantification and Data Analysis

Total RNA was extracted from cervical cells using a guanidine isothiocyanate lysis buffer as previously described [10]. The reverse transcription was performed using a High-Capacity cDNA Kit (Applied Biosystems, Waltham, MA, USA). and the transcript number of copies was measured by RT 5′exonuclease-PCR assay using the LightCycler 480 II sequence detector (Roche, Basel, Switzerland). RT PCR conditions, primer, and Taqman probe sequences for the type III IFNs genes, ISG15, TLR9, and TLR7 were previously described [10,33], the others were assayed on demand (IDT, Newark, NJ, USA): CCL20 (Hs.PT.58.19600309) and CCL5 (Hs.PT.58.1724551). A gene transcript was considered not detectable when the Ct value was >40. Target genes were normalized to the housekeeping beta-glucuronidase (GUS) because its expression is not influenced by HPV [10]. Accordingly, the difference in cycle thresholds (ΔCT) between GUS and the target genes was calculated for each gene in each cervical sample. These values were converted to relative expression values using 2^−ΔCT^. Relative expression values were log-transformed and analyzed using Spearman’s rank correlation coefficient (rho), with Kruskal–Wallis (KW) for difference among multiple groups and with Mann–Whitney (MW) U for each pairwise comparison using Bonferroni’s correction. The Jonckheere–Terpstra (JT) test, a rank-based nonparametric test, was also used to determine significant trends among ordered groups. The Pearson χ^2^-test was used for categorical variables. Statistical significance was set at *p* < 0.05. All statistical analyses were performed using IBM SPSS software, version 24.0.

## 3. Results

### 3.1. HPV, BV, and STI Distribution in the Study Group

From patients prospectively attending a cervicovaginal pathology unit, women were selected with stringent eligibility criteria, as described in the methods. A total of 120 women (median age 32 years, range 18–47) were enrolled, and they were stratified into ten-year age groups for analysis.

HPV DNA was detected in 51/120 (42.5%) cervical samples, and the HPV-positive patients were significantly (*p* = 0.001) younger (median age 30 y) than the HPV-negative (median age 35 y). The HPV genotypes (shown in Table 1 by age group) were 34/51 (66.7%) HR-HPVs. Specifically, 26/33 (78.8%) HR-genotypes belonged to the IARC group 1 and 8/33 (24.3%) to IARC groups 2A and B. The remaining 17 (33.3%) HPV-positive women had an LR-HPV infection (IARC group 3) (Table 1). The distribution of HPV test results (negative, positive to LR-HPV, and positive to HR-HPV), was significantly different (*p* = 0.007) among the three age groups (Table 1).

All samples were evaluated for the presence of BV by the Nugent score (BV-Nu), and in 111/120 unequivocal results were obtained: 59/111 (53.2%) samples were BV-Nu positive, while 52/111 (46.8%) were BV-Nu negative. The percentage of BV-Nu positivity did not differ among age groups but was significantly higher in HPV-positive women (*p* = 0.013).

The multiplex RT PCR assay performed to diagnose BV (BV-Rt) gave interpretable results in 116/120 patients: 30 (25.9%) resulted negative and 86 (74.1%) women were positive for BV-associated bacteria. According to the diagnostic criteria described in the methods section, 30 (25.9%) were classified as intermediate BV-Rt and 56 (48.3%) as full-positive BV-Rt. All subjects who were BV-Rt-negative (*n* = 30) were also negative for BV-Nu; of the remaining 22 BV-Nu-negative women, most (16/22: 72.7%) were referred to as intermediate BV-Rt and six as full-positive BV-Rt, whereas 12 BV-Nu-positive were intermediate BV-Rt (Table 2). Women were categorized as BV-negative (negative to microbiologic and molecular tests) and BV-positive in the subsequent analyses, the latter group corresponds to BV-Rt-intermediate and -positive women.

Bacterial species detected by the molecular assay are reported in Table 3. The presence of Lactobacillus spp. was not associated with the HPV status, whereas G. vaginalis and A. vaginae—the more common BV-associated bacteria found in 67.2% and 46.6% of tested samples—were more frequently detected in the HPV-positive than in the HPV-negative women (Table 3).

Results of the multiplex RT PCR panels for STI, obtained for 116 women, are shown in Table 4. Overall, 66/116 (56.4%) women were positive for at least one STI. The rate of STI positivity and the presence of any single microorganism were not significantly different among the age groups nor between the HPV-positive and the HPV-negative women. Contrastingly, BV-positive women were significantly more often to be STI-positive (*p* = 0.001, Table 4).

### 3.2. How Gene Expression Levels Vary According to HPV, BV, and STI Status

Expression levels of TLRs 7 and 9; of IFNL1, 2, 3, and IFNLR1, the specific subunit of their receptor; of ISG15; and CCL5 and CCL20 were measured in cervical cells from 120 enrolled women expression values were not detectable or had very low levels in 10 HPV-negative and two HPV-positive samples. Among 108 samples that gave measurable values, there was no relationship between patients’ age and the expression level of each studied gene (data not shown). Differently, a moderate positive association (Spearman’s rho range: 0.2 < r < 0.6 with *p*-values < 0.05) was found between levels of the TLR genes with all others, except for IFNLR1 and ISG15.

**Figure 1:**
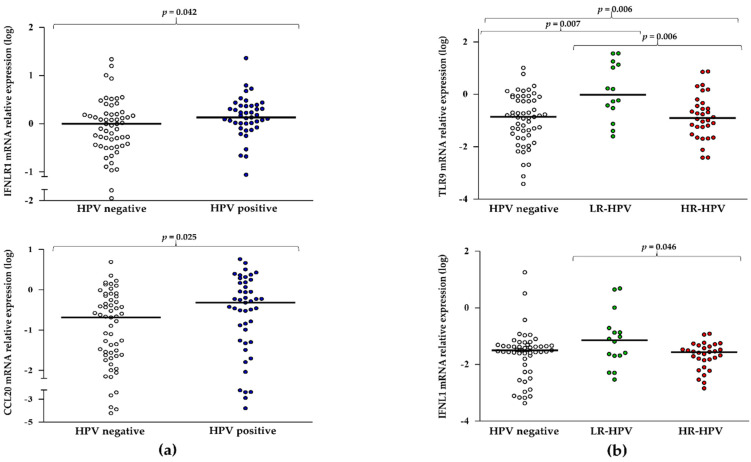
HPV-induced gene expression in cervical mucosa cells: (**a**) IFNLR1 and CCL20 in HPV-negative and HPV-positive women (**b**) TLR9 and IFNL1 in HPV-negative, LR-HPV, and HR-HPV-positive women. Relative mRNA expression values (*y*-axis), calculated using the threshold cycle relative quantification (2^−ΔCt^) and log-transformed, are reported. Horizontal lines indicate the median value of the group indicated below the *x*-axis. *P*-values were calculated using Mann–Whitney tests for comparisons between two groups and Kruskal–Wallis for comparisons between three groups.

Afterward, gene expression between 49 HPV-positive and 59 HPV-negative women was compared. No significant difference was found between the two groups in the expression of TLRs, IFNL1-3, ISG15, and CCL5 (Supplementary Figure S1), whereas that of IFNLR1 and CCL20 were significantly elevated in HPV-positive patients (MW *p* = 0.042 and *p* = 0.025, Figure 1a). Comparisons made with HPV-positive stratified in LR (*n* = 16) and HR (*n* = 33), evidenced activation of the expression of TLR9 (MW *p* = 0.006) in the LR-infected women, but not in the HR group (Figure 1b). Moreover, IFNL1 appeared to be expressed at higher levels in cervical cells positive to LR HPV compared with HR-HPV (MW *p* = 0.046, Figure 1b). No other significant difference in gene expression was observed among the groups (Supplementary Figure S2).

Comparing the expression of all study genes between women positive to the IARC carcinogenic group 1 HPV genotypes and the IARC groups 2A and B, no significant difference was found (data not shown).

Further analyses were conducted to dissect the impact of BV and STI on the activation of innate immune genes in response to HPV infections. Hence, gene expression results were evaluated by comparing a control group (i.e., women tested negative for HPV, BV, and STIs, *n* = 13) and the 49 HPV-positive samples stratified into three groups: positive to HPV and negative to BV and any STI (*n* = 7); positive to HPV and BV, negative to STI (*n* = 13); and positive to HPV and STIs (*n* = 29).

**Figure 2:**
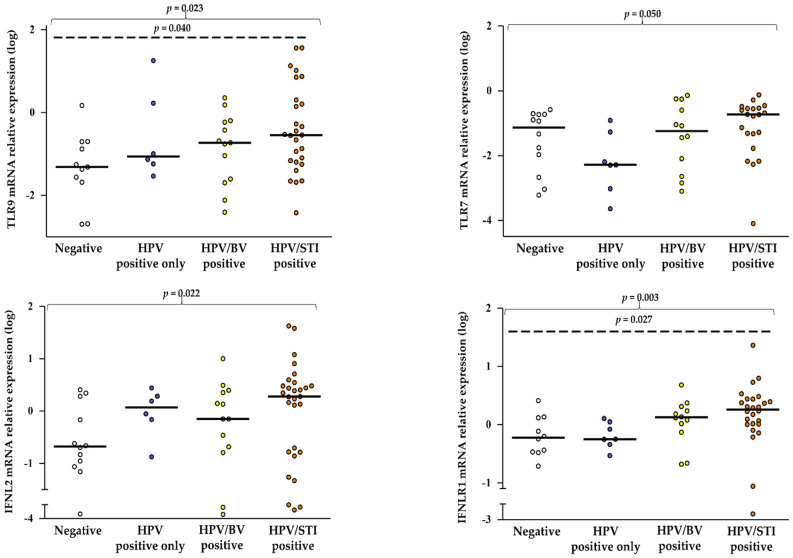
HPV infection induced different TLR9, TLR7, IFNL2, and IFNLR1 expressions in the presence of BV and STIs in cervical mucosa cells. Relative mRNA expression values (*y*-axis), calculated using the threshold cycle relative quantification (2^−ΔCt^) and log-transformed, are reported for the genes. Horizontal lines indicate the median value of the group indicated below the *x*-axis. *P*-values calculated using the Jonckheere–Terpstra (JT) test for trends among ordered groups are shown on top of each panel. *P*-values over the dotted lines refer to the comparison between the negative and the HPV/STI-positive women.

Levels of TLR9, TLR7, IFNL2, and IFNLR1 showed increasingly higher values from the control group to the positive to all conditions (JT test for trends: *p* = 0.023; *p* = 0.050; *p* = 0.022; *p* = 0.003, Figure 2), whereas the other values did not differ significantly (Supplementary Figure S3).

**Figure 3:**
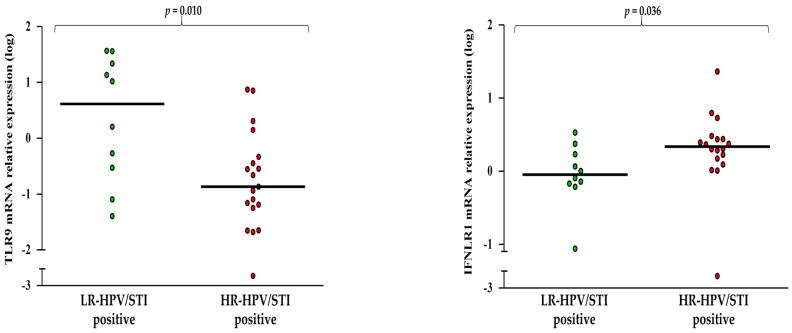
LR-HPV and HR-HPV induced significantly different TLR9 and IFNLR1 expressions in the presence of BV and STIs in cervical mucosa cells. Relative mRNA expression values (*y*-axis), calculated using the threshold cycle relative quantification (2^−ΔCt^) and log-transformed, are reported for the genes. Horizontal lines indicate the median value of the group indicated below the *x*-axis. *P* -values were calculated using Mann–Whitney tests for comparisons between two groups.

A further comparison was performed among women positive for HPV and STIs partitioning the LR-HPV (*n* = 10) and the HR-HPV groups (*n* = 19), which revealed significantly lower TLR9 (*p* = 0.010) and higher IFNLR1 (*p* = 0.036) values in the HR- with respect to the LR-positive samples (Figure 3 and Figure S4).

Finally, the contribution of specific bacteria to the expression of study genes, in groups of samples positive to only one BV-associated bacteria or only one STI compared with the control group, was analyzed but no significant differences were evidenced between groups for the very low number of women positive to only one microbe (data not shown).

## 4. Discussion

An efficient innate immune response is an essential step to avoid the persistence of HR HPV infections, the prerequisite for the development of cervical cancer. Among the action to counteract host immunity, HR HPV can inhibit TLR9-mediated activation of type I and III IFN response and cytokines’ production [4,34]. On the other hand, the host innate immune response is activated, but at different levels toward LR and HR HPVs and in transient versus persistent infections [7,8,10,33,35,36]. Studies conducted on cervical cells from naturally infected women have the potential to delineate activation of the immune response better than those conducted in HPV-transformed keratinocyte where the expression of the pro-oncogenic genes E6 and E7 is elevated and continuous [4,34]. Undoubtedly, the immune response to HPV is characterized by high individual variability [8,10,33] and may be affected by interactions with the cervicovaginal flora and, eventually, with other STIs.

Healthy vaginal flora is usually Lactobacillus-dominated whereas BV is associated with lower or absent Lactobacillus spp. and the presence of BV-associated bacteria [19,20]. BV was correlated with higher prevalence and persistence of HPV [26,30,37] and the main STIs [22,23]; the risk of these conditions was different among ethnic groups [27,38]. Fewer data were available on HPV prevalence in relation to the presence of BV-associated bacteria and of less pathogenic STIs not frequently diagnosed in a clinical context, such as *Ureoplasma* spp. and *M. hominis.* Moreover, studies focused on the impact that BV and STIs may have on the type III IFN response to HPV infections were missing.

To add more knowledge on the cervicovaginal mucosa innate immune response to HPV, a homogeneous group of sexually active Italian women of reproductive age was enrolled. Innate gene expression was measured in cervical cells in which HPV detection and genotyping, microbiologic and molecular diagnosis of BV, and molecular diagnosis of STIs were performed. More than 40% of the enrolled women were HPV-positive, even in the absence of cervical lesions, and more than 50% of the cases had BV and at least one STI, with a very high rate of co-presence of these two conditions. Women positive for HPV DNA were more frequently positive for BV (with microbiologic and with molecular diagnosis) and, particularly, for the two main bacterial species associated with BV: *G. vaginalis* and *A. vaginae*.

Despite individual variability, measured values of TLRs—which activate the mucosal innate immunity—were positively associated with those of type III IFN genes, and the studied cytokines. These associations point to a biological significance of measurements; therefore, some interesting conclusions can be drawn from these data.

The activation of TLR9 following HPV infections was detected in the LR, but much less in the HR-HPV-positive women, confirming our previous report [33]. This result is also in accordance with the downregulation of TLR9 expression, mediated by HPV16 E7 oncoprotein demonstrated in HPV-16-transformed cell lines [39]. Our result suggests that not only HPV16 but also the other HR-HPV genotypes were able to partially inhibit TLR9 expression; indeed, we found no differences in TLR9 expression between IARC group 1 and IARC group 2 A/B HPV-positive samples. Differently from TLR9, TLR7 expression was not coherently activated in the HPV-positive samples with the notion that TLR7 is mainly involved in RNA sensing. Nonetheless, high TRLR7 expression was a predictor of HPV clearance in women with cervical HPV infections [7,36]; unfortunately, follow-up samples were not available from our study group to confirm this association. IFNL1 was expressed at higher levels in cervical cells positive to LR HPV compared with HR HPV and HPV-negative cells, confirming our previous report [10] and suggesting a possible inhibitory activity of HR HPV toward IFNL1 expression. As regards chemokines’ expression, CCL20, but not CCL5, was activated in HPV-positive women independently from the oncogenic risk of the infecting genotypes and the presence of BV and STIs. A previous report [27] found that CCL20 (also named MIP-3α) was not associated with prevalent HPV infection or subsequent clearance; nonetheless, in a mouse model, papillomavirus-transformed dysplastic lesions were prevented by a functional CCR6/CCL20 axis [40].

Interesting considerations can be drawn regarding gene expression in the enrolled women stratified according to the HPV status and the presence of BV and other STIs, considering the lack of previous studies analyzing these conditions.

Increasingly elevated levels of TLR9 were observed in HPV-positive women with BV and STI, particularly in the LR HPV with bacterial co-infections. Upregulation in TLR9 transcription could be functional to clear viral and bacterial infection(s) but, paradoxically, TLR9 overexpression was previously linked to HPV persistence [33], high-grade lesions, and cancer [41,42,43]. Even though LR genotypes cause genital condylomas and are not related to cancer progression, infection persistence would be an unpleasant condition for women and their sexual partners. An increased expression of TLR7 was also observed in HPV-STI-positive women, but this new finding must be confirmed in prospective studies.

Our data showed that IFNL2 and, particularly, the type III IFN receptor-specific subunit, IFNLR1, could be driven at higher expression levels by BV and STIs. Cells of the female reproductive tract mucosa express IFNLR1 and thus respond to type III IFNs, a well-known first-line defense against viral infections in mucosal cells [44,45,46,47]. Indeed, type III IFNs can exert also harmful effects during viral and bacterial (co)infections; this complex scenario has been studied particularly in the digestive and respiratory tract. Type III IFNs may lower the efficacy of lung epithelial repair [48] and increase susceptibility to bacterial superinfections in the respiratory tract [45]. A well-calibrated binding affinity and different sensitivities to negative regulators of their receptor expression are part of the type I IFN response to avoid tissue damage, but these mechanisms are far less understood for type III IFNs [47,49]. In this complex scenario, it is known that type III IFNs bind with a higher affinity to IFNLR1 than to IL-10R2 (the other subunit), then mediate the antiviral response in the mucosa [47], but the mechanisms that regulate IFNLR1 expression are not yet elucidated [49]. In respiratory cells, our group detected elevated IFNLR1 expression in rhinovirus bronchiolitis in correlation with disease severity [50], but there are no previous in vivo studies conducted in cervical cells. Hence, further investigations are necessary to confirm the observation that a higher expression of IFNLR1 could be mediated by bacterial coinfections and whether this could influence HR HPV persistence.

Dysregulation in cytokine production was previously associated with HPV infection [27] and inflammatory conditions linked to BV and STIs [28]. However, the specific impact of the interplay of those microbial conditions on CCL5 and CCL20 production was not previously addressed. Our results point to activation of CCL20, but not of CCL5, in HPV infections, a response that seems not to be influenced by microbial coinfections. This is the first study reporting on the possible role of CCL20 in the mucosal immune response to HPV, despite the ability of HPV16 E6/E7 to inhibit its production in transformed keratinocytes [13].

This in vivo investigation was limited to a single-time-point investigation on the presence of HPV, BV, and common STIs, so it is impossible to infer whether BV creates an environment for the persistence of HPV infections, that in turn leads to a higher risk of STIs. Moreover, due to the limited number of samples, the differential impact of the different BV-associated bacteria, and specific STIs on the immune response to HPV could not be specifically evaluated. Therefore, large prospective studies with recurrent follow-up visits are needed to dissect the differential impact of a specific bacterial infection on the risk of HPV persistence.

In conclusion, high rates of prevalent HPV, BV, and several STIs not commonly diagnosed were found by molecular methods in Italian women of reproductive age attending a gynecological clinic for routine visits. Measurements of gene expression showed that STI-associated pathogens trigger TLR9 levels, possibly enhancing the risk of HPV persistence. Furthermore, elevated levels of the specific receptor IFNLR1 in HPV-, BV-, and STI- positive women point to a dysregulation of the type III IFN response that can act as a double-edged sword in infected epithelia. Hence, restoring a healthy vaginal microbiota could decrease HPV persistence and help clear other STIs, and consequently reduce invasive treatments and the progression to cervical cancer.

## Figures and Tables

**Table 1:** Results of human papillomavirus (HPV) detection and genotyping in women stratified by age.

Age Group	HPV NEG	LR HPV ^a^	HR HPV ^a^	*p*
18–27 (N = 35)	12 (34.3%)	8 (22.8%) 6 [1]; 44 [1]; 54 [1]; 61 [2]; 81 [2]; 84 [1]	15 (42.9%) 16 [3]; 18 [2]; 31 [1]; 33 [1]; 52 [1]: 53 [3]; 56 [1]; 58 [1]; 59 [1]; 66 [1]	0.007
28–37 (N = 57)	36 (63.1%)	5 (8.8%) 6 [2]; 11 [1]; 54 [1]; 84 [1]	16 (28.1%) 16 [5]; 31 [3]; 53 [1]; 58 [3]; 66 [2]; 68 [1]; 70 [1]	
38–47 (N = 28)	21 (75.0%)	4 (14.3%) 54 [2]; 61 [1]; 83 [1]	3 (10.7%) 16 [1]; 18 [1]; 58 [1]	

^a^ Human papillomavirus (HPV) genotypes were classified [2] as high-risk (HR, also including the probable HR 53 and 66) or low-risk (LR). The LR and HR genotypes detected are followed by square brackets in which the number of detections is reported. The *p*-value was calculated using the Pearson χ^2^-test.

**Table 2:** Results of bacterial vaginosis (BV) diagnosed by the Nugent score, human papillomavirus (HPV), and sexually transmitted infections (STI) detection in women stratified by BV molecular diagnosis (BV-Rt) results.

BV-Rt ^a^	BV-Nu ^b^ NEG	BV-Nu ^b^ POS	HPV NEG	HPV POS	STI NEG	STI POS
BV-Rt-negative	30	0	21	9	17	13
BV-Rt-intermediate	16	12	18	12	10	20
BV-Rt-full positive	6	47	27	29	13	43

^a^ Results of the molecular diagnosis of bacterial vaginosis (BV-Rt) are stratified into BV-Rt-negative, -intermediate, and -full positive according to the criteria described in materials and methods; ^b^ BV diagnosis obtained in 111 women with the Nugent score (BV-Nu) were considered negative (NEG) with scores < 7 or positive (POS) with scores 7–10.

**Table 3:** Distribution of the vaginosis-associated bacteria detected by the molecular assay with respect to human papillomavirus (HPV) results.

Vaginosis-Associated Bacteria ^a^		HPV		
		Negative	Positive	*p* Value
*Gardnerella vaginalis*	Negative	27	11	**0.045**
	Positive	39	39	
*Atopobium vaginae*	Negative	41	21	**0.039**
	Positive	25	29	
*Megasphaera type 1*	Negative	54	37	0.365
	Positive	12	13	
*Bacteroides fragilis*	Negative	63	50	0.258
	Positive	3	0	
*Mobiluncus* spp.	Negative	58	46	0.550
	Positive	8	4	
Bacterial vaginosis–associated bacteria 2	Negative	56	39	0.466
	Positive	10	11	

^a^ Bacteria were detected using the multiplex real-time (RT) PCR assays (Allplex™ STI/BV Panel Assays-Panel 4, Seegene) following the manufacturer’s instructions. The *p*-values were calculated using the Pearson χ^2^-test; significant *p*-values are in bold.

**Table 4:** Distribution of the sexually transmitted infections (STI) detected by the molecular assay with respect to human papillomavirus (HPV) and to bacterial vaginosis (BV) results.

Sexually Transmitted Infections ^a^		HPV		BV	
		Negative	Positive	Negative	Positive
*Ureaplasma parvum*	Negative	40	25	34	28
	Positive	26	25	18	30
*Ureaplasma urealiticum*	Negative	63	44	50	52
	Positive	3	6	2	6
*Mycoplasma genitalium*	Negative	65	49	51	58
	Positive	1	1	1	0
*Mycoplasma hominis*	Negative	57	44	46	51
	Positive	9	6	6	7
*Trichomonas vaginalis*	Negative	66	49	52	57
	Positive	0	1	0	1
*Chlamydia trachomatis*	Negative	63	47	52	52
	Positive	3	3	0	6
*Candida albicans/glabrata/krusei*	Negative	51	37	43	40
	Positive	15	13	9	18
All STIs	Negative	27	13	26	12
	Positive	39	37	26	46

^a^ Sexually transmitted infections were diagnosed using the multiplex real-time (RT) PCR assays (Allplex™ STI/BV Panel Assays 1 and 3, Seegene) following manufacturer’s instructions.

## Data Availability

The datasets containing all data analyzed, supporting the results of this study, will be made available by the authors, without undue reservation.

## Supplementary Materials

The following supporting information can be downloaded at: https://www.mdpi.com/article/10.3390/pathogens11111361/s1, Figure S1: gene expression levels of TLR7, TLR9, IFNL1, IFNL2, IFNL3, ISG15, and CCL5 in cervical mucosa cells of HPV-negative and HPV-positive women. Relative mRNA expression values (*y*-axis), calculated using the threshold cycle relative quantification (2^−ΔCt^) and log-transformed, are reported. Horizontal lines indicate the median value of the group indicated below the *x*-axis. All *p*-values calculated using Mann–Whitney tests for comparisons between two groups were >0.05. Figure S2: gene expression levels of TLR7, IFNL2, IFNL3, IFNLR1, ISG15, CCL5, and CCL20 in cervical mucosa cells of HPV-negative and HPV-positive women. Relative mRNA expression values (*y*-axis), calculated using the threshold cycle relative quantification (2^−ΔCt^) and log-transformed, are reported. Horizontal lines indicate the median value of the group indicated below the *x*-axis. All *p*-values calculated using Kruskal–Wallis tests for comparisons between three groups were >0.05. Figure S3: Gene expression levels of IFNL1, IFNL3, ISG15, CCL5, and CCL20 in cervical mucosa cells of negative, HPV-positive, HPV/BV-positive, and HPV/STI-positive women. Relative mRNA expression values (*y*-axis), calculated using the threshold cycle relative quantification (2^−ΔCt^) and log-transformed, are reported for the genes. Horizontal lines indicate the median value of the group indicated below the *x*-axis. All *p*-values calculated with the Jonckheere–Terpstra (JT) test for trends were >0.05. Figure S4: Gene expression levels of TLR7, IFNL1, IFNL2, IFNL3, ISG15, CCL5, and CCL20 in cervical mucosa cells of LR-HPV/STI-positive and HR-HPV/STI-positive women. Relative mRNA expression values (*y*-axis), calculated using the threshold cycle relative quantification (2^−ΔCt^) and log-transformed, are reported for the genes. Horizontal lines indicate the median value of the group indicated below the *x*-axis. All *p*-values calculated with Mann–Whitney tests for comparisons between two groups were >0.05.

## References

[1] Burchell A.N., Winer R.L., de Sanjosé S., Franco E. (2006). Chapter 6: Epidemiology and transmission dynamics of genital HPV infection. Vaccine.

[2] Muñoz N., Bosch F.X., de Sanjosé S., Herrero R., Castellsagué X., Shah K.V., Snijders P.J.F., Meijer C.J., International Agency for Research on Cancer Multicenter Cervical Cancer Study Group (2003). Epidemiologic Classification of Human Papillomavirus Types Associated with Cervical Cancer. N. Engl. J. Med..

[3] IARC Working Group (2012). IARC Monographs–100B: Human Papillomaviruses.

[4] Stanley M. (2021). Host defence and persistent human papillomavirus infection. Curr. Opin. Virol..

[5] Medzhitov R. (2001). Toll-like receptors and innate immunity. Nat. Rev. Immunol..

[6] McClure R., Emassari P. (2014). TLR-Dependent Human Mucosal Epithelial Cell Responses to Microbial Pathogens. Front. Immunol..

[7] Daud I.I., Scott M.E., Ma Y., Shiboski S., Farhat S., Moscicki A.-B. (2011). Association between toll-like receptor expression and human papillomavirus type 16 persistence. Int. J. Cancer.

[8] Pierangeli A., Degener A., Ferreri M., Riva E., Rizzo B., Turriziani O., Luciani S., Scagnolari C., Antonelli G. (2011). Interferon-Induced Gene Expression in Cervical Mucosa during Human Papillomavirus Infection. Int. J. Immunopathol. Pharmacol..

[9] Sommereyns C., Paul S., Staeheli P., Michiels T. (2008). IFN-Lambda (IFN-λ) Is Expressed in a Tissue-Dependent Fashion and Primarily Acts on Epithelial Cells In Vivo. PLOS Pathog..

[10] Cannella F., Scagnolari C., Selvaggi C., Stentella P., Recine N., Antonelli G., Pierangeli A. (2014). Interferon lambda 1 expression in cervical cells differs between low-risk and high-risk human papillomavirus-positive women. Med. Microbiol. Immunol..

[11] Chensue S.W. (2001). Molecular machinations: Chemokine signals in host-pathogen interactions. Clin. Microbiol. Rev..

[12] Cremel M., Berlier W., Hamzeh H., Cognasse F., Lawrence P., Genin C., Bernengo J.-C., Lambert C., Dieu-Nosjean M.-C., Delézay O. (2005). Characterization of CCL20 secretion by human epithelial vaginal cells: Involvement in Langerhans cell precursor attraction. J. Leukoc. Biol..

[13] Guess J.C., McCance D.J. (2005). Decreased migration of Langerhans precursor-like cells in response to human keratinocytes expressing human papillomavirus type 16 E6/E7 is related to reduced macrophage inflammatory protein-3alpha production. J. Virol..

[14] Jiang B., Xue M. (2015). Correlation of E6 and E7 levels in high-risk HPV16 type cervical lesions with CCL20 and Langerhans cells. Genet Mol. Res..

[15] Verteramo R., Pierangeli A., Mancini E., Calzolari E., Bucci M., Osborn J., Nicosia R., Chiarini F., Antonelli G., Degener A.M. (2009). Human Papillomaviruses and genital co-infections in gynaecological outpatients. BMC Infect. Dis..

[16] Sieving R.E., O’Brien J.R.G., Saftner M.A., Argo T.A. (2019). Sexually Transmitted Diseases Among US Adolescents and Young Adults: Patterns, Clinical Considerations, and Prevention. Nurs. Clin. N. Am..

[17] Ginocchio C.C., Chapin K., Smith J.S., Aslanzadeh J., Snook J., Hill C.S., Gaydos C.A. (2012). Prevalence of Trichomonas vaginalis and coinfection with Chlamydia trachomatis and Neisseria gonorrhoeae in the United States as determined by the Aptima Trichomonas vaginalis nucleic acid amplification assay. J. Clin. Microbiol..

[18] Leli C., Mencacci A., Latino M.A., Clerici P., Rassu M., Perito S., Castronari R., Pistoni E., Luciano E., De Maria D. (2017). Prevalence of cervical colonization by Ureaplasma parvum, Ureaplasma urealyticum, Mycoplasma hominis and Mycoplasma genitalium in childbearing age women by a commercially available multiplex real-time PCR: An Italian observational multicentre study. J. Microbiol. Immunol. Infect..

[19] Amabebe E., Anumba D.O.C. (2018). The Vaginal Microenvironment: The Physiologic Role of Lactobacilli. Front. Med..

[20] Fredricks D.N., Fiedler T.L., Marrazzo J.M. (2005). Molecular identification of bacteria associated with bacterial vaginosis. N. Engl. J. Med..

[21] Coudray M.S., Madhivanan P. (2019). Bacterial vaginosis—A brief synopsis of the literature. Eur. J. Obstet. Gynecol. Reprod. Biol..

[22] Cherpes T.L., Meyn L.A., Krohn M.A., Lurie J.G., Hillier S.L. (2003). Association between acquisition of herpes simplex virus type 2 in women and bacterial vaginosis. Clin. Infect. Dis..

[23] Masson L., Mlisana K., Little F., Werner L., Mkhize N.N., Ronacher K., Gamieldien H., Williamson C., Mckinnon L.R., Walzl G. (2014). Defining genital tract cytokine signatures of sexually transmitted infections and bacterial vaginosis in women at high risk of HIV infection: A cross-sectional study. Sex. Transm. Infect..

[24] Mao C., Hughes J.P., Kiviat N., Kuypers J., Lee S.-K., Adam D.E., Koutsky L.A. (2003). Clinical findings among young women with genital human papillomavirus infection. Am. J. Obstet. Gynecol..

[25] Nam K.H., Kim Y.T., Kim S.R., Kim S.W., Kim J.W., Lee M.K., Nam E.J., Jung Y.W. (2009). Association between bacterial vaginosis and cervical intraepithelial neoplasia. J. Gynecol. Oncol..

[26] Gillet E., Meys J.F., Verstraelen H., Bosire C., De Sutter P., Temmerman M., Broeck D.V. (2011). Bacterial vaginosis is associated with uterine cervical human papillomavirus infection: A meta-analysis. BMC Infect. Dis..

[27] Shannon B., Yi T.J., Perusini S., Gajer P., Ma B., Humphrys M.S., Thomas-Pavanel J., Chieza L., Janakiram P., Saunders M. (2017). Association of HPV infection and clearance with cervicovaginal immunology and the vaginal microbiota. Mucosal Immunol..

[28] Mitchell C., Marrazzo J. (2014). Bacterial vaginosis and the cervicovaginal immune response. Am. J. Reprod. Immunol..

[29] Torcia M.G. (2019). Interplay among Vaginal Microbiome, Immune Response and Sexually Transmitted Viral Infections. Int. J. Mol. Sci..

[30] Kero K., Rautava J., Syrjänen K., Grenman S. (2017). Association of asymptomatic bacterial vaginosis with persistence of female genital human papillomavirus infection. Eur. J. Clin. Microbiol..

[31] Usyk M., Zolnik C.P., Castle P.E., Porras C., Herrero R., Gradissimo A., Gonzalez P., Safaeian M., Schiffman M.R., Burk R.D. (2020). Cervicovaginal microbiome and natural history of HPV in a longitudinal study. PLOS Pathog..

[32] Nugent R.P., A Krohn M., Hillier S.L. (1991). Reliability of diagnosing bacterial vaginosis is improved by a standardized method of gram stain interpretation. J. Clin. Microbiol..

[33] Cannella F., Pierangeli A., Scagnolari C., Cacciotti G., Tranquilli G., Stentella P., Recine N., Antonelli G. (2015). TLR9 is expressed in human papillomavirus-positive cervical cells and is overexpressed in persistent infections. Immunobiology.

[34] Cigno I.L., Calati F., Albertini S., Gariglio M. (2020). Subversion of Host Innate Immunity by Human Papillomavirus Oncoproteins. Pathogens.

[35] Scott M.E., Ma Y., Farhat S., Moscicki A.-B. (2014). Expression of nucleic acid-sensing Toll-like receptors predicts HPV16 clearance associated with an E6-directed cell-mediated response. Int. J. Cancer.

[36] Halec G., Scott M.E., Farhat S., Darragh T.M., Moscicki A.-B. (2018). Toll-like receptors: Important immune checkpoints in the regression of cervical intra-epithelial neoplasia 2. Int. J. Cancer.

[37] O’Hanlon D.E., Gajer P., Brotman R.M., Ravel J. (2020). Asymptomatic Bacterial Vaginosis Is Associated with Depletion of Mature Superficial Cells Shed from the Vaginal Epithelium. Front. Cell Infect. Microbiol..

[38] Moscicki A.-B., Shi B., Huang H., Barnard E., Li H. (2020). Cervical-Vaginal Microbiome and Associated Cytokine Profiles in a Prospective Study of HPV 16 Acquisition, Persistence, and Clearance. Front. Cell Infect. Microbiol..

[39] Hasan U.A., Bates E., Takeshita F., Biliato A., Accardi R., Bouvard V., Mansour M., Vincent I., Gissmann L., Iftner T. (2007). TLR9 expression and function is abolished by the cervical cancer-associated human papillomavirus type 16. J. Immunol..

[40] Scagnolari C., Cannella F., Pierangelli A., Pilgrim R.M., Antonelli G., Rowley D., Wong M., Best S., Xing D., Roden R.B.S. (2020). Insights into the Role of Innate Immunity in Cervicovaginal Papillomavirus Infection from Studies Using Gene-Deficient Mice. J. Virol..

[41] Lee J.-W., Choi J.-J., Seo E.S., Kim M.J., Kim W.Y., Choi C.H., Kim T.-J., Kim B.-G., Song S.Y., Bae D.-S. (2007). Increased toll-like receptor 9 expression in cervical neoplasia. Mol. Carcinog..

[42] Hao Y., Yuan J.-L., Abudula A., Hasimu A., Kadeer N., Guo X. (2014). TLR9 expression in uterine cervical lesions of Uyghur women correlate with cervical cancer progression and selective silencing of human papillomavirus 16 E6 and E7 oncoproteins in vitro. Asian Pac. J. Cancer Prev..

[43] Baruah P., Bullenkamp J., Wilson P.O.G., Lee M., Kaski J.C., Dumitriu I.E. (2019). TLR9 Mediated Tumor-Stroma Interactions in Human Papilloma Virus (HPV)-Positive Head and Neck Squamous Cell Carcinoma Up-Regulate PD-L1 and PD-L2. Front. Immunol..

[44] Odendall C., Voak A.A., Kagan J.C. (2017). Type III IFNs are commonly induced by bacteria-sensing TLRs and reinforce epithelial barriers during infection. J. Immunol..

[45] Alphonse N., Dickenson R.E., Alrehaili A., Odendall C. (2022). Functions of IFNλs in Anti-Bacterial Immunity at Mucosal Barriers. Front. Immunol..

[46] Ank N., West H., Bartholdy C., Eriksson K., Thomsen A.R., Paludan S.R. (2006). Lambda interferon (IFN-lambda), a type III IFN, is induced by viruses and IFNs and displays potent antiviral activity against select virus infections in vivo. J. Virol..

[47] Lazear H.M., Schoggins J.W., Diamond M.S. (2019). Shared and Distinct Functions of Type I and Type III Interferons. Immunity.

[48] Broggi A., Granucci F., Zanoni I. (2019). Type III interferons: Balancing tissue tolerance and resistance to pathogen invasion. J. Exp. Med..

[49] Stanifer M.L., Pervolaraki K., Boulant S. (2019). Differential Regulation of Type I and Type III Interferon Signaling. Int. J. Mol. Sci..

[50] Pierangeli A., Statzu M., Nenna R., Santinelli L., Petrarca L., Frassanito A., Gentile M., Antonelli G., Midulla F., Scagnolari C. (2018). Interferon lambda receptor 1 (IFNL1R) transcript is highly expressed in rhinovirus bronchiolitis and correlates with disease severity. J. Clin. Virol..

