# Supplementary material for: Common Microbial Genital Infections and Their Impact on the Innate Immune Response to HPV in Cervical Cells
Source: Pathogens. 2022 Nov 16;11(11):1361. doi: 10.3390/pathogens11111361 (PMC9697853; doi:10.3390/pathogens11111361)
Supplement: Supplementary file 1 [file pathogens-11-01361-s001.zip › pathogens-1961658-supplementary.pdf]

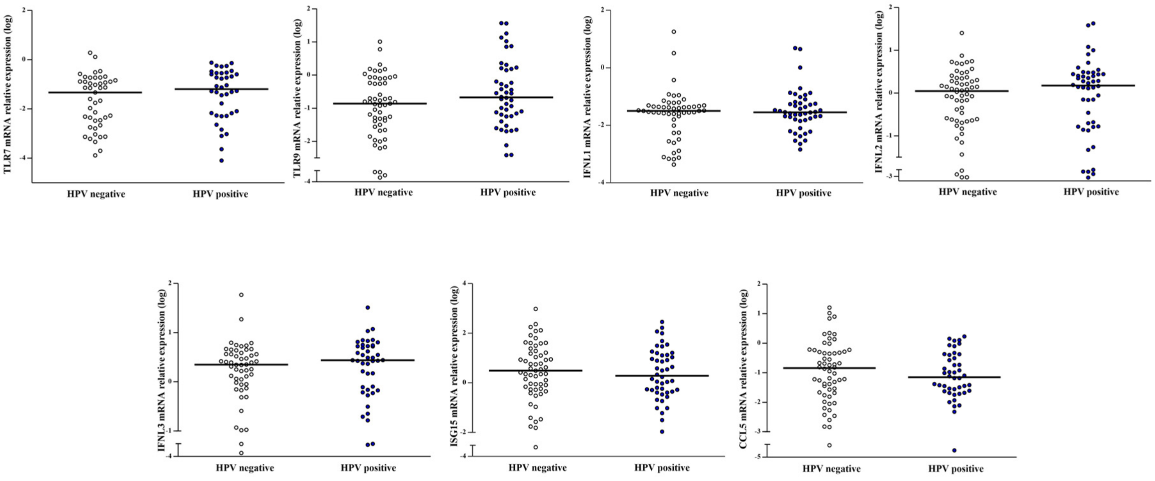

Figure S1: gene expression levels of TLR7, TLR9, IFNL1, IFNL2, IFNL3, ISG15, and CCL5 in cervical mucosa cells of HPV-negative and HPV-positive women. Relative mRNA expression values (*y*-axis), calculated using the threshold cycle relative quantification ($2^{-\Delta Ct}$) and log-transformed, are reported. Horizontal lines indicate the median value of the group indicated below the *x*-axis. All *p*-values calculated using Mann–Whitney tests for comparisons between two groups were >0.05.

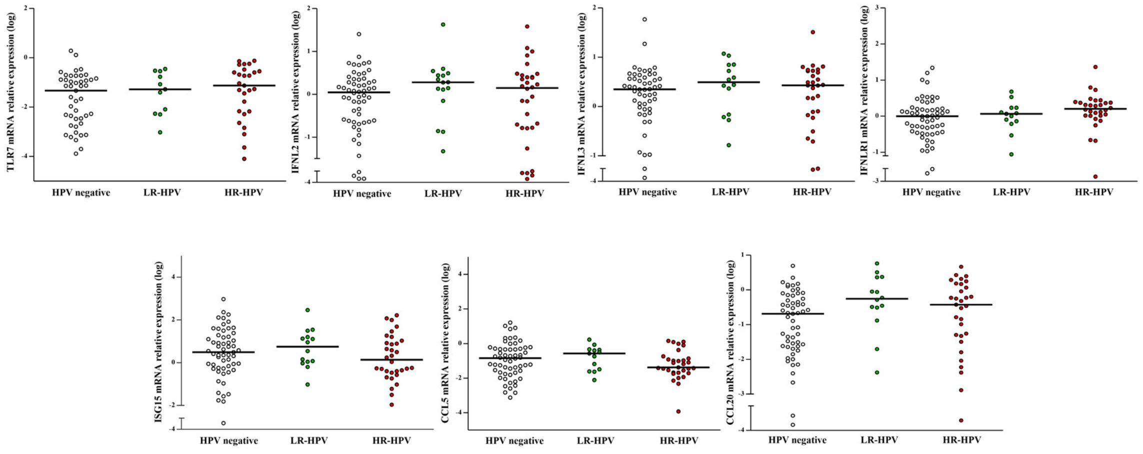

Figure S2: gene expression levels of TLR7, IFNL2, IFNL3, IFNLR1, ISG15, CCL5, and CCL20 in cervical mucosa cells of HPV-negative and HPV-positive women. Relative mRNA expression values (*y*-axis), calculated using the threshold cycle relative quantification ($2^{-\Delta Ct}$) and log-transformed, are reported. Horizontal lines indicate the median value of the group indicated below the *x*-axis. All *p*-values calculated using Kruskal–Wallis tests for comparisons between three groups were >0.05.

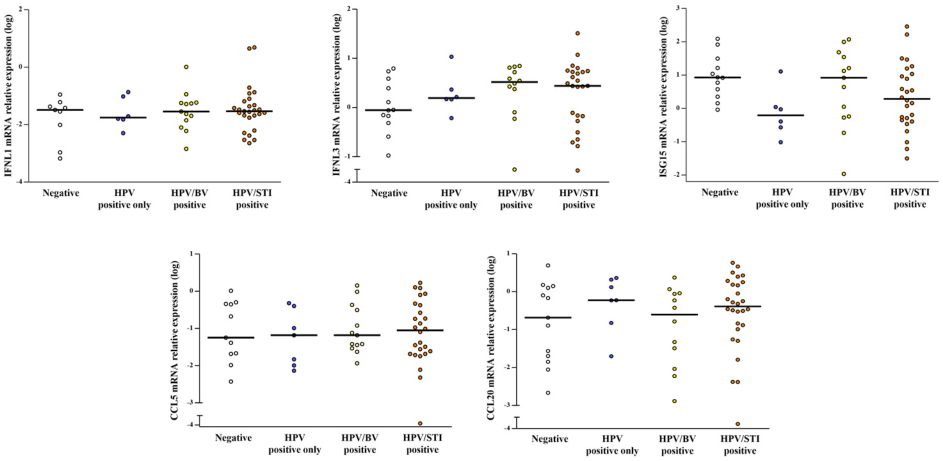

Figure S3: Gene expression levels of IFNL1, IFNL3, ISG15, CCL5, and CCL20 in cervical mucosa cells of negative, HPV-positive, HPV/BV-positive, and HPV/STI-positive women. Relative mRNA expression values (*y*-axis), calculated using the threshold cycle relative quantification ($2^{-\Delta Ct}$) and log-transformed, are reported for the genes. Horizontal lines indicate the median value of the group indicated below the *x*-axis. All *p*-values calculated with the Jonckheere–Terpstra (JT) test for trends were >0.05.

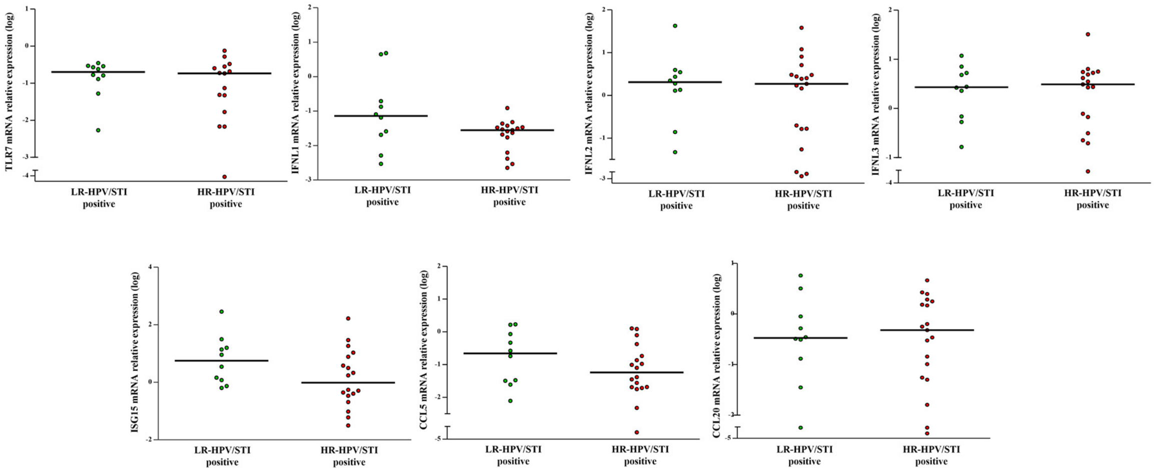

Figure S4: Gene expression levels of TLR7, IFNL1, IFNL2, IFNL3, ISG15, CCL5, and CCL20 in cervical mucosa cells of LR-HPV/STI-positive and HR-HPV/STI-positive women. Relative mRNA expression values (*y*-axis), calculated using the threshold cycle relative quantification ($2^{-\Delta Ct}$) and log-transformed, are reported for the genes. Horizontal lines indicate the median value of the group indicated below the *x*-axis. All *p*-values calculated with Mann–Whitney tests for comparisons between two groups were >0.05.
